# Pregnancy Promotes Maternal Hippocampal Neurogenesis in Guinea Pigs

**DOI:** 10.1155/2019/5765284

**Published:** 2019-04-11

**Authors:** Lily Wan, Tian Tu, Qi-Lei Zhang, Juan Jiang, Xiao-Xin Yan

**Affiliations:** Department of Anatomy and Neurobiology, Central South University Xiangya School of Medicine, Changsha, Hunan 410013, China

## Abstract

Adult neurogenesis in the hippocampal dentate gyrus (DG) modulates cognition and behavior in mammals, while motherhood is associated with cognitive and behavioral changes essential for the care of the young. In mice and rats, hippocampal neurogenesis is reported to be reduced or unchanged during pregnancy, with few data available from other species. In guinea pigs, pregnancy lasts ~9 weeks; we set to explore if hippocampal neurogenesis is altered in these animals, relative to gestational stages. Time-pregnant primigravidas (3-5 months old) and age-matched nonpregnant females were examined, with neurogenic potential evaluated via immunolabeling of Ki67, Sp8, doublecortin (DCX), and neuron-specific nuclear antigen (NeuN) combined with bromodeoxyuridine (BrdU) birth-dating. Relative to control, subgranular Ki67, Sp8, and DCX-immunoreactive (+) cells tended to increase from early gestation to postpartum and peaked at the late gestational stage. In BrdU pulse-chasing experiments in nonpregnant females surviving for different time points (2-120 days), BrdU+ cells in the DG colocalized with DCX partially from 2 to 42 days (most frequently at 14-30 days) and with NeuN increasingly from 14 to 120 days. In pregnant females that received BrdU at early, middle, and late gestational stages and survived for 42 days, the density of BrdU+ cells in the DG was mostly high in the late gestational group. The rates of BrdU/DCX and BrdU/NeuN colocalization were similar among these groups and comparable to those among the corresponding control group. Together, the findings suggest that pregnancy promotes maternal hippocampal neurogenesis in guinea pigs, at least among primigravidas.

## 1. Introduction

Neurogenesis occurs in the adult brain at particular localizations, as most frequently studied in the subventricular and the subgranular zones, wherein neural stem cells proliferate, differentiate, and mature into functional neurons [1–5]. Depending on species, the time course for newly produced neuroblasts to differentiate into mature neurons may take a few weeks to many months [3, 6–10]. Adult neurogenesis may provide a key substrate for brain structural plasticity that enables remodeling of neurocircuitry in response to internal and external stimuli and to fulfill new functional demands. For examples, adult hippocampal neurogenesis has been shown to play an essential role in spatial and contextual learning, new memory formation, and forgetting of some old memories [11–16]. Adult neurogenesis in the olfactory system is important for discrimination of and response to odorant signals [17–19]. Many other cognitive and behavioral functions have also been linked to olfactory and/or hippocampal adult neurogenesis, such as social and sexual interactions, offensive/defensive reactions, and depression or anxiety-like behaviors [20–29].

Motherhood is a dramatic life event in female and is associated with extensive neurobiological, cognitive, and behavioral changes involving multiple functional aspects such as learning and memory, development of mother-young bond, and nurture and anxious protection for the offspring [30–35]. These changes are facilitated by complex modulation in neuroanatomical, neurochemical, endocrinal, and immunological systems during pregnancy and after giving birth, ultimately to promote species survival and flourish [25, 36]. Reciprocal influences between maternal reproductive experience and neuroplasticity in various brain regions have been demonstrated or proposed in animal and human studies [31, 37–45].

Given its critical role in brain structural plasticity [2–5, 16], one can expect that adult neurogenesis could be altered during pregnancy, thereby participating in motherhood-related neuroplasticity [46, 47]. Small rodents specifically mice and rats have been mostly used to address this issue. Cell proliferation in the subventricular zone is reported to be increased at certain gestational (GD) and postpartum (PD) days in mice (GD7 and PD7) and rats (GD21), but unchanged at other examined time points, i.e., GD14, PD0, PD14, and PD21 in mice and GD7, PD2, PD8, and PD28 in rats [48–50]. An increased cytogenesis may give rise to new neurons in the olfactory bulb that facilitate odor-based cognitive activity and behavior [36, 51–53]. In the DG, cell proliferation at the SGZ remains unchanged at GD7 but appears reduced at other gestational time points (such as GD14.5, GD16.5, and GD18.5) in mice [54, 55]. However, in rats, no alteration in cell division in the SGZ is observed during gestation [36, 56–58]. Sheep is another mammal that has been investigated in context of adult neurogenesis relative to maternal experience [10]. Specifically, there is a decrease in cell proliferation in the DG in mothers staying with their lamb during early postpartum period, while it remains unclear whether hippocampal neurogenesis is changed during pregnancy [59]. Taking these existing data together, it appears that pregnancy may influence adult neurogenesis in a manner that is related to animal species and gestational stage, as well as the location of neurogenic niche.

Guinea pigs appear fairly unique among rodents or even the majority of small- to medium-sized mammals by an impressively long duration of gestation, 63–68 days in average (https://en.wikipedia.org/wiki/List_of_mammalian_gestation_durations). Guinea pigs are also interesting in relation to motherhood because they give birth to precocial young, whereas most other rodents are born altricially. They are used as experimental models to address some scientific questions related to reproduction, fetal growth, and neurodevelopment [60–65]. In the present study, we set to explore whether adult hippocampal neurogenesis is altered in guinea pigs during pregnancy, especially relative to gestational stages. Experiments were carried out using first-time pregnant mothers (primigravidas) and age-matched nonpregnant females as control. The first set of experiments was designated to determine neurogenic potential via examination of the amount of immunolabeling (+) of endogenous cellular markers including Ki67, Sp8, and doublecortin (DCX) in the dentate gyrus (DG) of primigravidas at early gestational, late gestational, postpartal, and postweaning stages, relative to nonpregnant females. The second set of experiments was carried out to estimate the time frame of neuronal differentiation of newly produced cells in the SGZ in nonpregnant females, using bromodeoxyuridine (BrdU) birth-dating combined with double immunofluorescent labeling for DCX and the neuron-specific nuclear antigen (NeuN). This led to the third set of experiments to verify the change in cell proliferation in the DG and neurogenic potential thereof at early, middle and late gestational stages in pregnant animals relative to the control group, assessed at 42 days post BrdU administration.

## 2. Materials and Methods

### 2.1. Animals and Experimental Design

Male (used for breeding) and female Hartley guinea pigs were obtained from and maintained in the Center for Laboratory Animals of Xiangya School of Medicine during all experimental procedures before perfusion. Animals were housed in temperature, humidity and lighting (12 h/12 h light-on and off) controlled vivarium with free access to standard laboratory chow and water, with fruit or fresh vegetable also provided daily. The use of animals was in compliance with the National Institutes of Health Guide for the care and use of laboratory animals. All experimental procedures were approved by the Ethics Committee of Xiangya School of Medicine.

Timed pregnancy was initiated by co-housing a virgin female at 3 months of age with an adult male (~6 month-old). The female was checked every morning for the presence of vaginal plug, and once found, the animal was recorded as being pregnant at gestational day 1 and the male was removed from the mating cage. The female was further monitored for signs of pregnancy (weight gain and abdominal enlargement). Other virgin females were not allowed for mating, and served as age-matched non-pregnant controls.

For the first experiment, pregnant females were perfused at the end of the third gestational week (GW) (defined as early gestation, *n* = 3), at the end of GW7 (defined as late gestation, *n* = 3), at the end of the second postpartal week (PPW2, *n* = 5), and at the end of the fourth postpartal week (PPW4, *n* = 4, considered as a postweaning stage), respectively. For the latter two groups, pups were stayed together with the mother until the end of the second week. Nonpregnant females at 3, 4, and 5 months of age (*n* = 3/age point) were included in the control group. For the second experiment, nonpregnant females were injected with BrdU (Sigma-Aldrich, St. Louis, MO, USA; B5002, i.p., 50 mg/kg, twice with an 8-hour interval) when they were 3 months old and allowed to survive for 2 (*n* = 4), 7 (*n* = 4), 14 (*n* = 4), 30 (*n* = 4), 42 (*n* = 4), 60 (*n* = 4), and 120 (*n* = 4) days post-BrdU injection. For the third experiment, pregnant females were injected with BrdU (i.p., 50 mg/kg, twice with an 8-hour interval) at the last 1 or 2 day of GW2 (*n* = 4), GW5 (*n* = 5), and GW8 (*n* = 4). All of these animals were perfused after they survived for 42 days following BrdU administration.

### 2.2. Tissue Processing

At the designated time points during pregnancy, after giving birth, or post-BrdU injections, animals were perfused via the ascending aorta with 4% paraformaldehyde in 0.01 M phosphate-buffered saline (pH 7.4, PBS) under overdose anesthesia (sodium pentobarbital, 100 mg/kg, i.p.). The whole brain was removed from the cranium, postfixed in the perfusion fixative for 2 days. The brains were transferred into 30% sucrose for cryoprotection. The cerebrum was cut frontally in a cryostat at 30 *μ*m, with 24 sets of sections passing the rostrocaudal dimension of the hippocampus collected in PBS serially in cell culture plates. Thus, each well contained equally distant (~30 × 24 = 720 *μ*m) sections from the septal to temporooccipital levels of the hippocampus from both hemispheres.

### 2.3. Immunohistochemistry

From each brain, separate sets of hippocampal sections were immunohistochemically stained with the avidin-biotin complex (ABC) method using previous characterized commercial primary antibodies to visualize labeling of Ki67, Sp8, DCX, and BrdU. Ki67 is an endogenous nuclear marker expressed in the S phase of cell cycle therefore reflecting active cell proliferation [66]. Sp8 is an endogenous transcription factor expressed in putative neuronal precursors in the subventricular and subgranular zones [66]. DCX is a known endogenous marker for immature neurons [66, 67]. BrdU is a thymidine analog that can be incorporated into DNA during cell division thereby serving as an exogenous tracing marker of cell proliferation and subsequent differentiation and maturation [68]. Sections were treated first with 3% hydrogen peroxide (H_2_O_2_) in PBS for 30 minutes (min), and preincubated in 5% normal horse (for Ki67, Sp8, and DCX stains) or rabbit (BrdU stain) serum in PBS with 0.3% Triton X-100 for 1 hour (h) at room temperature, to lower nonspecific reactivity. Specifically for BrdU immunolabeling, sections were additionally treated in 1× SSC and 50% formamide for 1 h at 65°C, then in 2 N HCl for 30 min at 37°C, before H_2_O_2_ bleaching. Sections were then incubated, respectively, with rabbit anti-Ki67 (Vector Lab., Burlingame, CA, USA; #014-1107, diluted at 1 : 2000), goat anti-Sp8 (Santa Cruz Biotech Inc., CA, USA; sc-104661, 1 : 1000), goat anti-DCX (Santa Cruz Biotech.; sc-8066, 1 : 2000), and rat anti-BrdU (Bio-Rad Lab. Inc., Hercules, CA, USA; MCA2060, 1 : 2000) overnight at 4°C with gentle agitation on a rotator. Sections were further incubated in PBS containing biotinylated universal secondary antibody (house anti-mouse, rabbit, and goat IgGs) or rabbit anti-rat IgG at 1 : 400 for 2 h and subsequently with freshly prepared avidin-biotin mixer (1 : 400) (Vector Lab.) for 1 h. Immunoreaction in sections was developed in PBS containing 0.003% H_2_O_2_ and 0.05% diaminobenzidine (DAB). Three 10-minute washes with PBS were used between the incubations. The immunolabeled sections were mounted on slides, allowed to air-dry, lightly counterstained with hematoxylin, and coverslippered after dehydration and clearance through ascending ethanol and xylene.

### 2.4. Double Immunofluorescence

Double immunofluorescence was carried for confocal microscopic analysis of BrdU/DCX and BrdU/NeuN colocalization. A set of hippocampal sections from each BrdU injected animal was pretreated in 1× SSC and 50% formamide for 1 h at 65°C and in 2 N HCl for 30 min at 37°C for antigen retrieval, followed by incubation in PBS containing 5% donkey serum and 0.3% Triton X-100 for 1 h for lowering nonspecific labeling. The sections were further reacted with rat anti-BrdU (1 : 2000) together with goat anti-DCX (1 : 1000), or rat anti-BrdU together with mouse anti-NeuN (Merck-Millipore, MAB377, 1 : 2000) overnight at 4°C. On the next day, the sections were washed a few times with PBS and reacted for 2 h at room temperature with Alexa Fluor® 488- and Alexa Fluor® 594-conjugated secondary antibodies (Jackson ImmunoResearch Laboratories Inc., West Grove, PA, USA; 1 : 200). Finally, the sections were counterstained with bisbenzimide (i.e., Hoechst 33342, Sigma-Aldrich, St. Louis, MO, USA; B2883, 1 : 50000). After rinsing with PBS, the sections were coverslippered with antifading medium before microscopic examination.

### 2.5. Microscopic Imaging and Cell Count

Immunolabeled sections were examined and imaged on an Olympus fluorescent light microscope (BX53) equipped with a digital imaging system (cellSens Standard, Olympus, Japan) and a Nikon confocal fluorescent microscope (Nikon, Digital Eclipse C1 plus). For sections immunolabeled with DAB as the chromogen, the entire area of DG was imaged using 2× objective for orientation and measurement of the length of the granule cell layer (GCL). Then, images were taken using 4× and 10× objectives by moving along the GCL by centering the microscopic field over the SGZ. These images were used to quantify the amounts of Ki67+, Sp8+, DCX+, and BrdU+ cells in the dorsal part of the DG. Images captured as the above were subjected to cell count, calculation of relative density, and colocalization frequency. For all measurements, the area of interest (AOI) was defined as a band region equal to the thickness of the GCL but centered over the SGZ. To achieve the above, the image was opened with the Photoshop software; two lines were drawn along the upper and low borders of GCL in reference to hematoxylin or bisbenzimide counterstain. Then, this line template was moved towards the hilus by a half of the distance of thickness of the GCL. By enlarging the images on the computer screen, the numbers of Ki67+, Sp8+, DCX+, and BrdU+ cells within the above AOI were counted, with the sums calculated for each hippocampal section. The length of GCL in the same section was measured by referring to the scale bar. Finally, the cell density, expressed as number of cells per unit (mm) length of GCL, was calculated for each brain, using the data obtained from the 2nd to 5th equally spaced (rostral-to-caudal direction) hippocampal sections from both hemispheres (the first section at the septal end had no or only a small cross-sectional area of the DG and therefore not included for quantification). It should be also noted that we used the dorsal hippocampus for quantification for a consideration of keeping the sampling region consistent as well as the greater extent of adult neurogenesis in the dorsal than ventral hippocampal regions in most mammals [69–71].

For BrdU/DCX and BrdU/NeuN double immunofluorescence, four midhippocampus sections from the two hemispheres of each brain were used for quantitative analysis. Low-magnification images over the entire DG were taken with 4× objective on the BX53 microscope for the counting of the total number of BrdU+ cells along the SGZ. The sections were then examined on the Nikon confocal fluorescent microscope. While examining the section at 10x to 20x magnifications, cells appeared to show colocalization were imaged, with colocalization verified following overlying of the single-channel images. Cells exhibiting DCX or NeuN colocalization were counted in each section, with the sum calculated for each brain. Overall, there were ~50 BrdU+ cells per section in average; therefore, a total of ~200 BrdU+ cells per brain were included in the analysis of the colocalization rate for each pair of double immunofluorescence.

### 2.6. Data Assembly, Statistical Analysis, and Figure Preparation

Cell counting was carried out by experimenters who were blinded to the animal grouping information, with the data gathered by the primary experimenter for quantitative and statistical analyses. Mean numeric density (number of cells/mm GCL length) and colocalization rate (% colocalized) were calculated in Excel spreadsheets for individual animals. Data from individual animals were entered into Prism spreadsheets (GraphPad Prism 4.1, San Diego, CA) under corresponding groups, with graphs prepared using box-plot illustration. Statistical analyses were conducted using a nonparametric test (Kruskal-Wallis with Dunn's multiple comparison test), with *P* value and Kruskal-Wallis statistic index (briefed as KWS) reported. Statistically significant difference was considered when *P* < 0.05. Figures were assembled with Photoshop 7.1 and converted into TIFF files, with contrast/brightness in the entire panel adjusted as needed.

## 3. Results

### 3.1. Increased Ki67+ Cells in the DG of Pregnant Relative to Nonpregnant Animals

**Figure 1:**
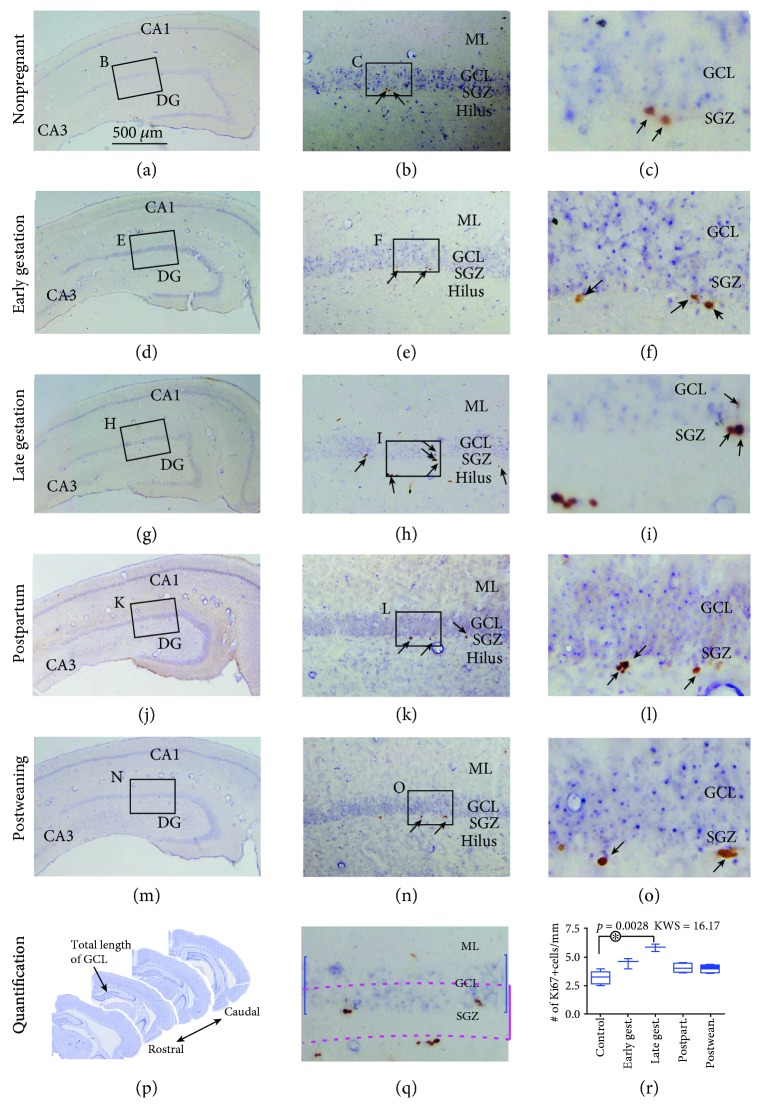
Characterization of cytogenetic activity with the cell cycle marker Ki67 in time-pregnant guinea pigs at early gestational, late gestational, postpartal, and postweaning stages relative to age-matched nonpregnant females. The pregnant animals are at the end of the third (GW3) and seventh (GW7) weeks of gestation and at the end of the second (PPW2) and fourth (PPW4) weeks postpartum, respectively. Representative images of Ki67 immunolabeling (brown) with hematoxylin counterstain (blue) from individual animal groups are illustrated as indicated. The left panels (a, d, j, and m) are low-magnification images showing the orientation of Ammon's horn (CA1, CA3) and dentate gyrus (DG), with framed areas enlarged as the middle panels, in which boxed areas are further enlarged as the right panels. Ki67 immunoreactive (+) profiles (pointed by arrows) are nuclear in nature and localized at the subgranular zone (SGZ), with some occurring in clusters (i, l, and o). (p) shows the rostrocaudal range of hippocampal sections in each brain used to quantify Ki67+ cells in the dorsal part of the DG. (q) shows the designation of a band area for counting Ki67+ cells along the granule cell layer (GCL). This band is defined to have a height that equals to the depth (blue line) of the GCL but is moved towards the hilus by a half of the GCL thickness (purple lines). (r) plots the numeric densities of Ki67+ cells, expressed as number (#) of cells per mm of GCL length, in the groups. There is a significant difference of the medians in the groups by Kruskal-Wallis test, with that between the control and late gestational groups reaching significance (^∗^) by post hoc analysis. KWS: Kruskal-Wallis scientific index. Scale bar = 500 *μ*m in (a) applying to (d, g, j, and m), equal to 100 *μ*m for (b, e, h, k, n, and q), and 25 *μ*m for (c, f, i, l, and o).

Ki67 is a nuclear antigen expressed during the S phase of cell cycle, which can be used to mark actively proliferating neural stem cells or neuroblasts in the brain [67, 72]. Consistent with the specific site of hippocampal adult neurogenesis, Ki67+ cells were observed but essentially located along the SGZ of the DG in all animals examined (Figures 1(a)–1(o)). At high magnifications, the labeled profiles were seen small in size consistent with a localization of the immunoreactivity to the cell nuclei. These Ki67+ nuclei also tended to occur in pairs or small clusters at the SGZ (Figures 1(f), 1(i), 1(l), and 1(o)).

Quantification was carried out by counting the number of labeled cells along the SGZ over the band region with its height equivalent to the depth of the GCL in equally spaced rostral to caudal hippocampal sections (Figures 1(p) and 1(q)) (also applying to the measurements for other labeling below). Thus, the numeric densities, expressed as (median (interquartiles), same format below), of subgranular Ki67+ cells were 3.3 (1.0), 4.7 (0.8), 5.9 (0.6), 4.2 (0.8), and 4.1 (0.5) cells/mm of GCL length in the nonpregnant control, early gestational, late gestational, postpartal, and postweaning groups, respectively. Kruskal-Wallis test showed a significant difference of the medians among the groups (*P* = 0.0028, KWS = 16.17). However, post hoc Dunn's multiple comparison test reported a statistically significant difference between the control and the late gestational groups, but not for the remaining intercomparison groups (Figure 1(r)).

### 3.2. Increased Sp8+ Cells in the DG of Pregnant Relative to Nonpregnant Animals

Sp8 is a zinc finger transcription factor involved in interneuron formation [73–76]. It is richly expressed in the subcortical ganglionic eminence and the SVZ during brain development [73]. In various adult mammals, it marks interneurons in the olfactory bulb, striatum, and cerebrum [77–79]. A Drosophila study has showed its expression in intermediate neural progenitors that appears to help maintain the population of neural stem cells [80]. Our recent study has identified Sp8 expression in putative neural progenitor cells in both the SVZ and SGZ in guinea pigs and human [66].

**Figure 2:**
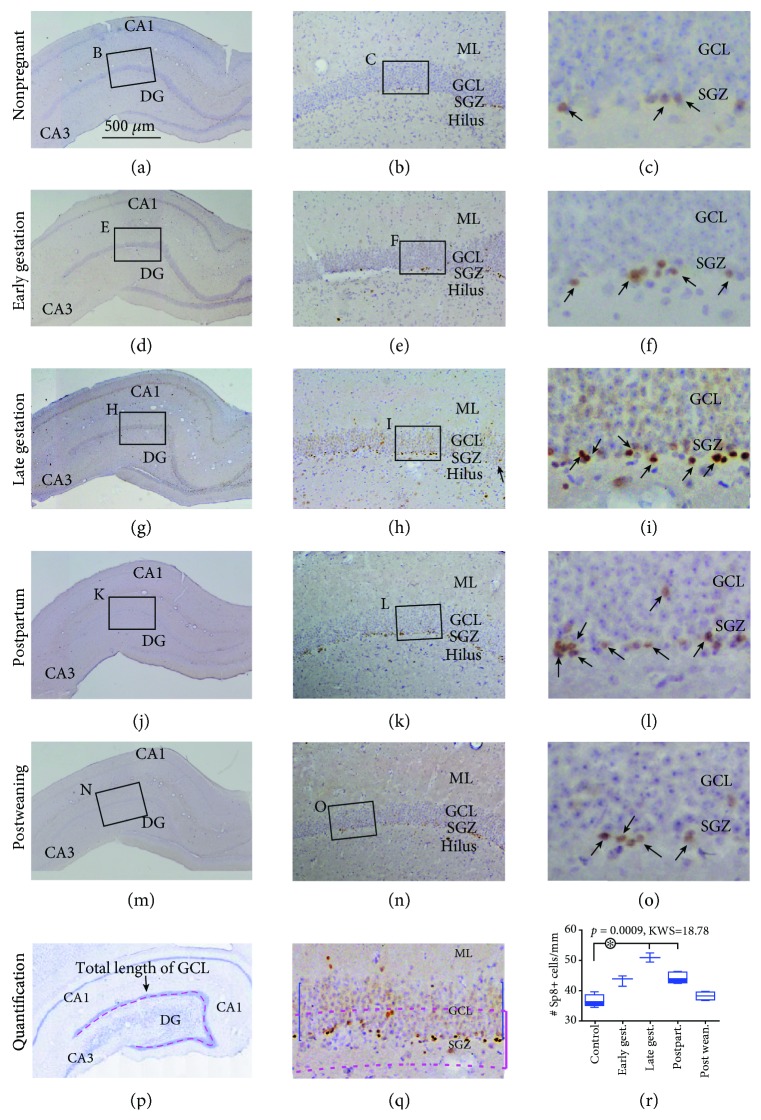
Analysis of putative neural precursors with immunolabeling of the zinc finger transcription factor Sp8 in guinea pigs at early gestational, late gestational, postpartal, and postweaning stages relative to nonpregnant females. Midhippocampal images of Sp8 immunolabeling (brown) with hematoxylin counterstain (blue) from individual animal groups are illustrated as indicated. The left panels (a, d, j, and m) are low-magnification images showing the orientation of hippocampal formation, with framed areas enlarged accordingly as the middle (b, r, h, k, and n) and right (c, f, i, l, and o) panels. Sp8+ profiles (pointed by arrows) are nuclear as judged by the size and shape in reference to hematoxylin stain and are overwhelmingly localized to the SGZ (b, c, e, f, h, i, k, l, n, and o). The labeled cells are noticeably denser in the dentate gyrus of the late gestational group (i) than in other groups (c, f, l, and o). (p) illustrates the method for measuring the total length of the GCL. (q) shows the designation of a band area for counting Sp8+ cells along the GCL (the same as noted for Ki67+ cells in Figure 1). (r) plots the numeric densities of Sp8+ cells in the groups. There is a significant difference of the medians in the groups by Kruskal-Wallis test. Post hoc analysis reports significant difference (^∗^) for late gestational and postpartal groups relative to the control group. Abbreviations are as defined in Figure 1. Scale bar = 500 *μ*m in (a) applying to (d, g, j, and m), equal to 250 *μ*m for (p), 100 *μ*m for (b, e, h, k, n, and q) and 25 *μ*m for (c, f, i, l, and o).

A considerable population of Sp8+ cells occurred in the DG over the SGZ in the pregnant and control groups (Figures 2(a)–2(o)). Sp8 immunolabeling was localized to cell nuclei based on the size of labeled profiles in reference to the hematoxylin-stained nuclei (Figures 2(b), 2(c), 2(e), 2(f), 2(h), 2(i), 2(k), 2(l), 2(n), and 2(o)). However, Sp8+ labeled cells in the SGZ did not exhibit an apparent clustered distribution as seen for Ki67+ cells. In general, the Sp8+ cells in the SGZ showed intense labeling, while there were also a smaller number of lightly labeled cells in the middle of the GCL (Figures 2(c), 2(f), 2(i), 2(l), and 2(o)). By visual inspection of the light microscopic images, it appeared that the labeled cells were mostly dense in the late gestational relative to other groups (Figures 2(b), 2(e), 2(h), 2(k), and 2(n)). Using the same quantification method as described above for the Ki67+ cells, the numeric densities of Sp8+ cells over the SGZ region were estimated for the groups (Figures 2(p) and 2(q)). The densities of subgranular Sp8+ cells in the nonpregnant control, early gestational, late gestational, postpartal, and postweaning groups were 35.8 (3.3), 43.9 (3.4), 50.4 (3.0), 43.6 (3.0), and 37.8 (2.5) cells/mm, respectively. There was a significant difference of the medians among the groups (*P* = 0.0009, KWS = 18.78). Post hoc analysis indicated a statistically significant difference between the control and the late gestational groups and between the control and the postpartal groups as well (Figure 2(r)).

### 3.3. Increased DCX+ Cells in the DG of Pregnant Relative to Nonpregnant Animals

**Figure 3:**
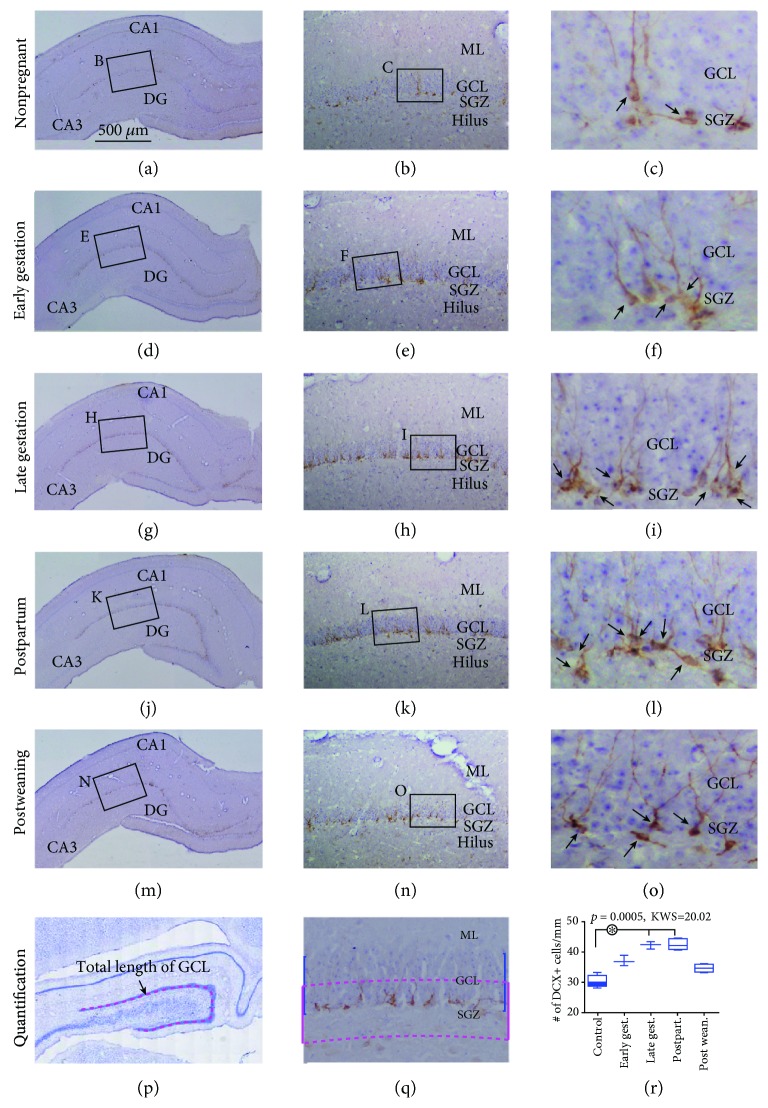
Characterization of hippocampal immature neurons with immunolabeling of doublecortin (DCX) in guinea pigs at early gestational, late gestational, postpartal, and postweaning stages relative to nonpregnant females. DCX+ neurons (brown) with hematoxylin counterstain (blue) from individual animal groups are as indicated, with low-magnification images as left panels (a, d, j, and m) and enlarged views as the middle (b, e, h, k, and n) and right (c, f, i, l, and o) panels. DCX+ immature neurons (pointed by arrows) are overwhelmingly localized to the SGZ, with labeled dendritic processes extending into the molecular layer (ML) (b, c, e, f, h, i, k, l, n, and o). The amount of labeled cells appears greater in the late gestational and postpartal groups (i, l) than in other groups (c, f, and o). (p) illustrates the measuring of the total length of the GCL. (q) shows the measuring template for counting DCX+ cells over the subgranular zone. (r) plots the numeric densities of DCX+ cells in the groups. There is a significant difference of the medians in the groups by Kruskal-Wallis test, with significant difference (^∗^) for the late gestational and postpartal groups in comparison to the control group by post hoc test. Abbreviations are as defined in Figure 1. Scale bar = 500 *μ*m in (a) applying to (d, g, j, and m), equal to 250 *μ*m for (p), 100 *μ*m for (b, e, h, k, n, and q), and 25 *μ*m for (c, f, i, l, and o).

DCX+ cells in the DG showed characteristic morphology of immature neurons, with immunolabeling clearly displaying the somatic and dendritic processes (Figures 3(a)–3(o)). The amounts of labeled cells appeared to be denser in the early gestational, late gestational, and postpartal groups relative to the nonpregnant and postweaning groups by closer inspection of micrographs (Figures 3(b), 3(e), 3(h), 3(k), and 3(n)). Quantification of DCX+ cells over the SGZ of the dorsal DG was carried out for the individual groups (Figures 3(p) and 3(q)). The densities of DCX+ cells were 30.0 (3.5), 37.2 (2.8), 42.8 (2.2), 42.7 (3.2), and 35.2 (2.1) cells/mm in the nonpregnant, early gestational, late gestational, postpartal, and postweaning groups, respectively. The Kruskal-Wallis test indicated that the medians varied significant among the groups (*P* = 0.0005, KWS = 20.02). Post hoc analysis indicated significant differences for the control relative to the late gestational groups and for the control relative to the postpartal groups (Figure 3(r)).

### 3.4. Increased BrdU+ Cells in the DG of Pregnant Relative to Nonpregnant Animals

**Figure 4:**
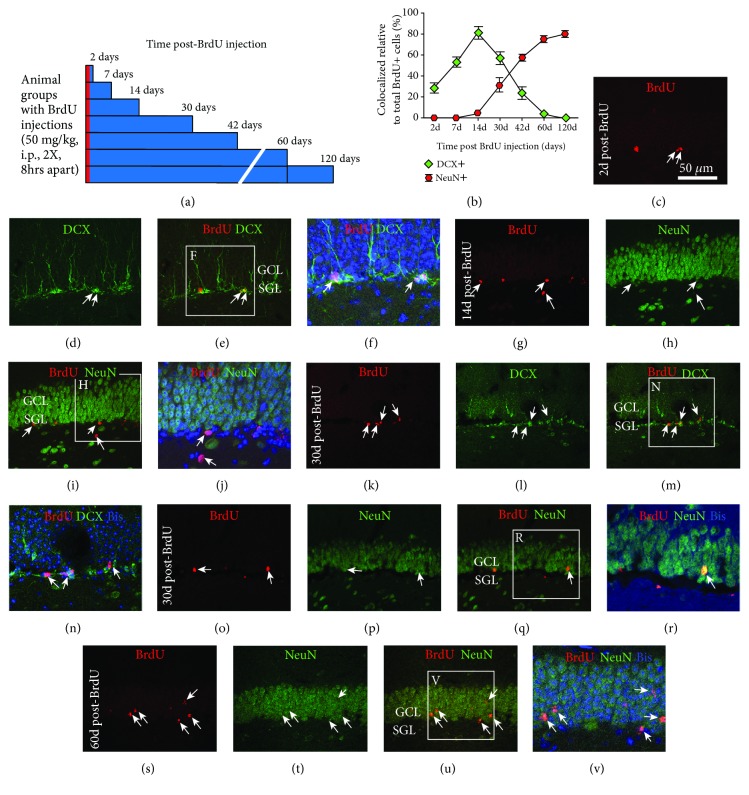
Assessment of the time course of neuronal differentiation of newly produced subgranular cells in adult female guinea pigs with BrdU pulse-chasing. (a) illustrates the experimental design. Two BrdU injections are given to nonpregnant animals at 3 months of age, with brains examined at 2, 7, 14, 30, 42, 60, and 120 days post-BrdU administration. (b) summarizes the percentage rates of BrdU+ cells colocalizing with doublecortin (DCX) and the neuronal nuclear antigen (NeuN) at the above surviving time points based on confocal microscopic analysis. BrdU birth-dated cells coexpress DCX as early as 2 days up to 42 days and coexpress NeuN from 14 days onward. A transition from DCX- to NeuN-expressing granule cells appears to occur largely between 30 to 42 days post-BrdU incorporation. (c–v) show representative confocal double immunofluorescent images at the surviving time points as indicated, with BrdU labeling in red, DCX and NeuN labeling in green, and bisbenzimide (Bis) nuclear labeling in blue. Arrows point to cells colabeled for BrdU and DCX or NeuN. Abbreviations are as defined in Figure 1. Scale bar = 50 *μ*m in (c) applying to (d, e, g–i, k–m, o–q, and s–u) and equal to 25 *μ*m for the far right image panels.

Following the above assessment of neurogenic potential with endogenous markers, we attempted to confirm an increased hippocampal neurogenesis during pregnancy with the BrdU birth-dating method. However, an initial uncertain issue was the appropriate surviving time point to be used in adult guinea pigs following BrdU injection, which was not available from literature. It was considered that besides exogenously labeling newly produced cells, the time point should also allow an assessment of the trend of neurogenic differentiation of these cells. Therefore, we first carried out a BrdU pulse-chasing study in nonpregnant females, with BrdU injections given to a group of animals at ~3 months of age, followed by brain examination at multiple surviving time points, i.e., 2, 7, 14, 30, 42, 60, and 120 days, post-BrdU application (Figure 4(a)). The extent to which BrdU+ cells colocalizing with the immature neuronal marker DCX and the mature neuronal marker NeuN was determined (Figure 4(b)).

On microscopic examination, nuclear profiles with bright BrdU immunofluorescence were observed in the DG in all animals that received BrdU injections (Figures 4(c), 4(g), 4(k), 4(o) and 4(s)) but were not detected in parallel-processed sections from animals that did not received BrdU injection (data not shown). In animals surviving 2-30 days, BrdU+ cells were located at or close to the SGZ, while there was a trend of BrdU+ cells to reside in the GCL in animals surviving 30, 42, 60, and 120 days (Figure 4(s)). BrdU+ cells were found to colocalize with DCX in animal groups surviving 2-42 days, but not in the groups surviving longer. In contrast, BrdU+ cells were rarely found to colocalize with NeuN in groups surviving 2, 7, and 14 days but did so in the remaining groups with longer surviving times. These observations are shown as representative sets of double immunofluorescent images (Figures 4(c)–4(v)). Quantitatively, the rates (median (interquartiles) %) of BrdU+ cells coexpressing DCX were 28.3 (4.9), 53.5 (3.6), 82.0 (6.1), 57.3 (7.1), 23.7 (5.5), and 2.5 (1.8) for the animal groups surviving 2, 7, 14, 30, 42, and 60 days, whereas no colocalization was found in animals surviving 120 days. The rates of BrdU+ cells coexpressing NeuN were 3.5 (2.8), 31.3 (7.0), 59.2 (4.1), 76.8 (3.7), and 82 (3.3) for the animal groups surviving 14, 30, 42, 60, and 120 days and zero for the groups surviving 2 and 7 days (Figure 4(b)).

**Figure 5:**
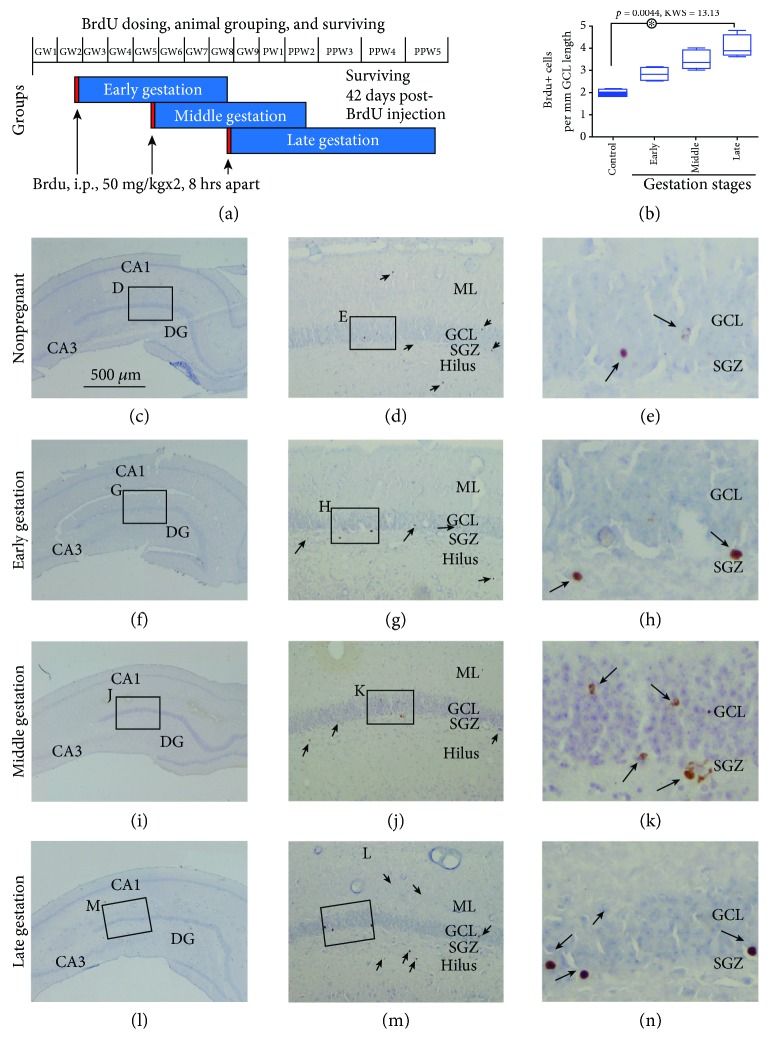
Assessment of the amounts of newly formed hippocampal dentate cells in pregnant guinea pigs at early, middle, and late gestational stages relative to nonpregnant controls at 42 days after BrdU incorporation. (a) illustrates the experimental design. Two BrdU injections are given to time-pregnant animals at the end of the second, fifth, and eighth week of gestation, with brains examined 42 days after BrdU administration. Brains from the nonpregnant group surviving for 42 days after BrdU injection (as noted in Figure 4) are examined as controls. (b) plots the numeric densities of BrdU+ cells obtained from the groups, which are quantified using the same method for Ki67, Sp8, and DCX labeling as shown in Figures 1–3. There exists a trend of increase in BrdU+ cells over the three gestational stages relative to control, with the difference between the late gestational and control groups reaching significance. Representative images of BrdU labeling (brown) with hematoxylin counterstain (blue) from individual groups are shown at low magnification and enlarged views, as indicated (c–l). BrdU+ nuclear profiles are indicated by arrows. Abbreviations are as defined in Figure 1. Scale bar = 500 *μ*m in (c) applying to (f, i, and l);, equivalent to 100 *μ*m for (d, g, j, and m), and 25 *μ*m for (e, h, k, and n).

According to the above data, we chose 42 days as the surviving time for the animal groups designated to receive BrdU administration at their early, middle, and late trimesters of gestation (Figures 5(a) and 5(b)). By using this time point (rather than 30 days that appeared also reasonable), the terminal examination would be after giving birth for the animals receiving BrdU pulse-chasing at and after middle gestation. This could allow a cross-validation of the timing of BrdU injection through an extrapolation in reference to the date of giving birth. BrdU+ cells appearing as nuclear profiles were observed in the DG of all animals, localizing to the SGZ as well as in the middle of the GCL (Figures 5(c)–5(l)). In closer examination, most cells exhibited fairly condensed immunoreactivity, while some of them appeared granular, especially those residing in the GCL (Figures 5(e), 5(h), 5(k) and 5(l)). Cell clusters were infrequently observed, almost always at the SGZ (Figures 5(j) and 5(k)). Quantification was carried out to estimate the numeric density of BrdU+ cells relative to the length of the GCL using the same method as described above for the Ki67+, Sp8+, and DCX+ cells. The comparing groups included the three groups that received BrdU during pregnancy as well as the nonpregnant group surviving 42 days post-BrdU injection. The numeric densities of BrdU+ cells were 2.0 (0.3) (median (interquartiles)), 2.8 (0.6), 3.4 (0.9), and 3.9 (0.9) cells/mm of GCL length in the nonpregnant control, early gestational, middle gestational, and late gestational groups, respectively. Thus, there was a trend of increase of BrdU+ cells from early to late gestational stages, with the Kruskal-Wallis test showing a significant difference of the medians among the groups (*P* = 0.0044, KWS = 13.13). However, post hoc Dunn's multiple comparison test reported that the difference reached significance between the control and the late gestational groups, but not between other individually comparing groups (Figure 5(b)).

### 3.5. Neuronal Differentiation of BrdU+ Cells in Pregnant and Nonpregnant Animals

**Figure 6:**
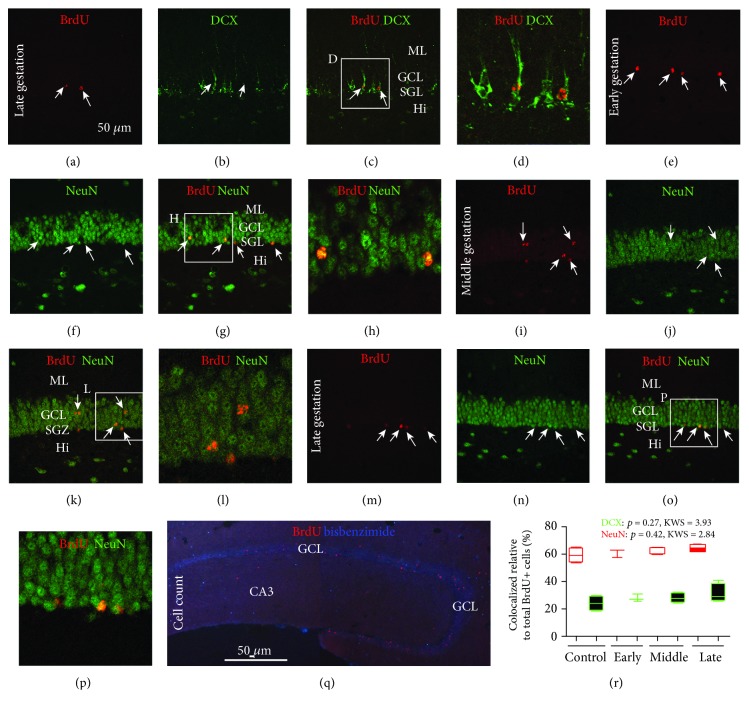
Verification of a neurogenic potential of hippocampal dentate cells formed at early, middle, and late gestational stages in pregnant guinea pigs. Colocalization of DCX and NeuN with BrdU is assessed at 42 days after BrdU incorporation, with data from a control group surviving for the same time point included. (a–p) show examples of BrdU/DCX (a–d) and BrdU/NeuN (e–p) double-labeled cells in the dente gyrus from the animal groups as indicated. BrdU/DCX coexpressing cells have well-developed dendritic processes (a–p). BrdU reactivity can appear granular in BrdU/NeuN colabeled granule cells (e–l). (q) shows the method for counting the total number of BrdU+ cells along the GCL of the dorsal hippocampus. (r) plots the rates of colocalization quantified in animals from the four groups. There is no difference between the groups regarding the percentage rate of BrdU+ cells coexpressing DCX or NeuN, according to the results of Kruskal-Wallis test as indicated. Abbreviations are as defined in Figure 1. Scale bar = 50 *μ*m in (a) applying to (b, c, e–g, i–k, m–o) and equivalent to 25 *μ*m for (d, h, l, and p) and the bar in (q) equals 500 *μ*m.

To verify that BrdU pulse-chased cells in the DG at early, middle, and late gestational stages would undergo neuronal differentiation, microscopic examination and quantification for BrdU/DCX and BrdU/NeuN double immunofluorescence were carried out in hippocampal sections from the above animals surviving 42 days post-BrdU injection. BrdU+ cells at the SGZ and GCL were found to colocalize with DCX and with NeuN in all these animal groups (Figures 6(a)–6(q)). In BrdU/DCX colocalized granule cells, the dendritic processes exhibited clear immunofluorescence and extended across the GCL into the molecular layer (Figures 6(a)–6(d)). In many BrdU/NeuN colocalized cells, the BrdU immunolabeling appeared granule-like in the nuclei (Figures 5(e)–5(p)). Cell counts were carried out to estimate the percentage rates of BrdU+ cells coexpressing DCX or NeuN in the three gestational groups, with data obtained from the nonpregnant group surviving 42 days post-BrdU injection serving as control. Among the total number of BrdU+ cells counted over the anatomically defined SGZ band (Figure 6(q)), the rates (median (interquartiles) %) for those colocalizing with DCX were 24.2 (9.4), 27.4 (4.6), 28.0 (7.6), and 29.5 (12) for the nonpregnant, early gestational, middle gestation, and late gestational groups, respectively. The rates for BrdU+ cells colocalizing with NeuN were 60.9 (9.2), 63.1 (5.1), 64.1 (4.2), and 64.3 (4.6) for the groups as listed above in the same order. There were no statistical differences between the comparing groups either for the rates of BrdU+ cells colocalizing with DCX (*P* = 0.27, KWS = 3.93), or for the rates of BrdU+ cells with NeuN (*P* = 0.42, KWS = 2.84) (Figure 6(r)).

## 4. Discussion

Neuroplasticity in association with pregnancy and postpartal experience consists of an important subject of research in reproductive neurobiology, which may be relevant to mental health in humans even at old ages [36, 81–83]. Although the basic hormonal and neuroendocrine mechanisms regulating reproduction would be very likely conserved through evolution, there exist remarkable differences between mammalian species or even substrains of the same species regarding motherhood (or more generally parenthood) behavior [25, 33, 84–90]. Therefore, it is important to explore basic issues of neuroplasticity in partnership with pregnancy, such as adult neurogenesis, in multiple animal species. Here, we show an increased hippocampal neurogenesis in pregnant guinea pigs and that the newly formed neurons could differentiate into mature-like granule cells.

In the current study, we examine the neurogenic potential with three endogenous molecular markers Ki67, Sp8, and DCX that reflect active cell proliferation, population of putative neuronal precursors, and immature neurons [66, 67, 91], respectively, in the DG of primigravidas at early gestational (GW3), late gestational (GW7), postpartal (PPW2), and postweaning (PPW4) stages, relative to age-matched nonpregnant females. The numeric densities of Ki67+, Sp8+, and DCX+ cells in the dentate neurogenic zone show a trend of increase in the pregnant groups relative to the control group. Using the nonparametric Kruskal-Wallis test (a relatively robust statistical test), a significant difference is found for the cells labeled by these markers between the late gestational and control groups. Therefore, these data suggest an increased neurogenic potential during pregnancy especially around the late stage of gestation in guinea pigs. Consistent with this notion, there is a trend of increase in BrdU+ cells in the DG pulse-chased at the end of the 2nd, 5th, and 8th week of gestation in pregnant relative to nonpregnant female, with the effect reaching statistical significance in the late gestational group. Together, the findings obtained via assessments of endogenous and exogenous cellular markers support a conclusion that in guinea pigs, pregnancy promotes adult (maternal) hippocampal neurogenesis.

As noted in Introduction, in mice and rats, hippocampal neurogenesis is unchanged [50, 56–58, 86] or reduced [55, 86] during pregnancy. Therefore, guinea pigs are different from mice and rats in the context of the influence of pregnancy on adult hippocampal neurogenesis. The precise reason underlying the observed difference between guinea pigs and mice/rat remains unknown. However, in general, substantial species differences in adult neurogenesis exist between mammals [3–5]. Being considered a rodent, guinea pigs show other remarkable differences from mice and rats in perspectives of reproductive behavior, cellular elements facilitating cerebral neuroplasticity [92], and even phylogenetic molecular biology [93, 94]. Besides a much longer duration of pregnancy, guinea pig mother gives birth to fewer pups than mice and rats, with the newborns well-developed morphologically and locomotively [95], unlike the offspring of most rodents that are born altricially. Relevant to adult cerebral neuroplasticity [96–98], DCX+ immature neurons are present in layer II of the neocortex and amygdalar complex in adult guinea pigs, as with many larger mammals including humans [67, 91, 99–102], but different from mice and rats in which these cells appear to present essentially in the paleocortex [103]. In addition to the above, we show here that the time course of neuronal differentiation of adult-born subgranular cells in adult guinea pigs is different relative to mice and rats as well as larger mammals as reported in literature. Based on the frequencies of BrdU/DCX and BrdU/NeuN colocalization following cell birth-dating, it takes approximately two months for most newly generated subgranular cells to become NeuN-expressing mature-like granule cells in guinea pigs. Notably, in mice and rats, this process occurs in about 3 weeks [6, 104], while functional integration of the adult-born cells into neuronal circuitry may take place over a longer period [1, 14]. In sheep, it takes about 4 months for most BrdU pulse-chased subgranular cells to differentiate into mature-like granule cells that express NeuN [105]. Further, in nonhuman primates, postmitotic differentiation of adult-born granule cells protracts over even a longer time period. Thus, the expression of DCX in these cells exceeds six months in macaque monkeys [7].

Our BrdU birth-dating study in pregnant guinea pigs indicates that by 6 weeks (42 days surviving time point), newly generated hippocampal dentate neurons can express DCX as well as NeuN (which is comparable to those seen in nonpregnant females). According to previous characterizations [1, 98], cells with this mixed neurochemical property are likely at a transitional stage of morphological differentiation into maturing functional neurons. Importantly, these transitional neurons undergo dynamic neuritic extension, synaptogenesis, network formation, and modification in orchestrating environmental stimulation [14]. Therefore, these findings indicate that hippocampal neurons generated from the middle to late gestational stages not only can survive but are undergoing the process of differentiation towards maturing granule cells during the postpartal period. Specifically, given that these granule cells exhibit a mixed neurochemical feature characteristic of a transitional status of neuronal development in the postpartal guinea pig mothers, the differentiation and maturation of these young neurons could be also influenced by motherhood activities such as mother-young bonding and nurturing and caring of the offspring.

Future studies are needed to further explore the alteration of adult hippocampal neurogenesis during pregnancy among mammalian species, as well as the regulatory mechanism and implications thereof. For instances, adult neurogenesis, cognitive functions, and behavior are related to or modified by age and likely motherhood experience [3, 25, 106–109]. Because our experiments were carried out in primigravidas at young adulthood, it remains unknown if such an effect exists in multiparous females. Pregnancy and motherhood are supported by complex endocrinal modulations, with the levels of multiple hormones (e.g., estrogen, progesterone, chorionic gonadotropin, prolactin, adrenal cortisol, and thyroxine) elevated during pregnancy and postpartum in a stage-dependent manner. Previous studies using pharmacological and certain experimental (e.g., ovariectomy) manipulations have suggested that some of the above hormones can individually stimulate or inhibit adult hippocampal neurogenesis in rodents [110–115]. However, it is obviously challenging or impossible to separately define the action of individual hormones during a natural course of pregnancy. Therefore, the current finding of adult neurogenesis change with pregnancy in guinea pigs should be interpreted as a sum or synergetic effort of hormonal regulations.

In summary, via characterization of cell proliferation and neuronal differentiation with endogenous immunogenic markers and BrdU birth-dating technology, the present study provides evidence for an increased hippocampal neurogenesis in pregnant guinea pigs, especially at the late gestational stage among the primigravidas. Newly generated dentate granule cells in young adult females exhibit largely an immature neuronal phenotype for at least 6 weeks. The granule cells produced during the middle to late gestational period are expected to be integrated into functional neuronal networks under the influence of postpartal motherhood experience.

## Data Availability

The data used to support the findings of this study are available from the corresponding author upon request.

## Additional Points

*Highlights*. Reproductive experiences may modulate neuroplasticity, cognition, and behavior. Pregnancy lasts approximately 9 weeks in guinea pigs. The maturation time of new hippocampal neurons takes ~2 months in guinea pigs. Neurogenic potential is increased during pregnancy in guinea pigs. New hippocampal neurons differentiate during the postpartal period.
